# Neuropsychiatric symptoms and social cognition in frontotemporal dementia and Alzheimer’s disease

**DOI:** 10.1590/1980-5764-DN-2025-0409

**Published:** 2026-06-15

**Authors:** Lucas de Andrade Saraiva, Elisa de Paula França Resende, Luciano Inácio Mariano, Danielly Silva Paula, Paulo Caramelli, João Vinícius Salgado, Leonardo Cruz de Souza

**Affiliations:** 1 Universidade Federal de Minas Gerais, Faculdade de Medicina, Grupo de Pesquisa em Neurologia Cognitiva e do Comportamento, Belo Horizonte MG, Brazil.; 2 Universidade Federal de Minas Gerais, Faculdade de Medicina, Departamento de Psiquiatria, Belo Horizonte MG, Brazil.; 3 Universidade Federal de Minas Gerais, Instituto de Ciências Biológicas, Programa de Pós-Graduação em Neurociências, Belo Horizonte MG, Brazil.; 4 Faculdade de Ciências Médicas de Minas Gerais, Belo Horizonte MG, Brazil.; 5 Universidade Federal de Minas Gerais, Faculdade de Medicina, Departamento de Clínica Médica, Belo Horizonte MG, Brazil.; 6 Universidade Federal de Minas Gerais, Instituto de Ciências Biológicas, Departamento de Morfologia, Belo Horizonte MG, Brazil.

**Keywords:** Frontotemporal Dementia, Alzheimer disease, Psychic Symptoms, Social Cognition, Demência Frontotemporal, Doença de Alzheimer, Sintomas Psíquicos, Cognição Social

## Abstract

Neuropsychiatric symptoms (NPS) are common in dementia and may hinder differential diagnosis between behavioral variant frontotemporal dementia (bvFTD) and Alzheimer’s disease (AD). Despite investigations of NPS and social cognition, particularly Theory of Mind (ToM) and emotion recognition, their precise, differential impact on social cognition in bvFTD versus AD remains less characterized.

**Objective:**

This study examined NPS in bvFTD and their association with social cognition, comparing results to AD.

**Methods:**

Patients with bvFTD (n=13) and AD (n=18) were assessed using neuropsychiatric and cognitive batteries measuring depression, anxiety, mania, and psychosis. Social cognition was evaluated with the Mini-Social and Emotional Assessment (mini-SEA), including the *Faux-Pas* test (ToM) and the Facial Emotion Recognition Test (FERT).

**Results:**

The groups did not differ in sex, education, or disease duration. Compared to AD, bvFTD patients had higher manic symptoms (p<0.001) and schizophrenia spectrum symptoms, particularly in negative (p<0.001) and resistance/hostility (p=0.034) symptoms. In bvFTD, depressive (p=0.020) and anxious (p=0.045) symptoms positively correlated with the *Faux-Pas* test, while positive psychotic symptoms correlated negatively (p=0.005). In AD, obsessive-compulsive (p=0.013) and disorganized psychotic symptoms (p=0.009) negatively correlated with the *Faux-Pas* test, while manic symptoms negatively correlated with the FERT (p=0.033).

**Conclusion:**

bvFTD patients displayed more schizophrenic and manic symptoms than AD, with NPS impacting social cognition differently in each condition.

## RESUMO

Sintomas neuropsiquiátricos (SNP) são comuns na demência e podem dificultar o diagnóstico diferencial entre a variante comportamental da demência frontotemporal (DFTvc) e a doença de Alzheimer (DA). Embora os SNP e a cognição social — especificamente a Teoria da Mente (ToM) e o reconhecimento de emoções — tenham sido investigados, o impacto distinto desses SNP na cognição social, na DFTvc em comparação com a DA, permanece pouco caracterizado.

**Objetivo:**

Este estudo examinou os SNP na DFTvc e sua associação com a cognição social, comparando os resultados com a DA.

**Métodos:**

Pacientes com DFTvc (n=13) e DA (n=18) foram avaliados por meio de baterias neuropsiquiátricas e cognitivas que mensuraram depressão, ansiedade, mania e psicose. A cognição social foi avaliada com a *Mini-Social and Emotional Assessment* (mini-SEA), incluindo o teste de *Faux-Pas* (ToM) e o Teste de Reconhecimento de Emoções Faciais (FERT).

**Resultados:**

Os grupos não diferiram quanto a sexo, escolaridade ou duração da doença. Em comparação à DA, os pacientes com DFTvc apresentaram sintomas maníacos mais elevados (p<0,001) e sintomas do espectro da esquizofrenia, particularmente sintomas negativos (p<0,001) e de resistência/hostilidade (p=0,034). Na DFTvc, sintomas depressivos (p=0,020) e ansiosos (p=0,045) correlacionaram-se positivamente com o teste de *Faux-Pas*, enquanto sintomas psicóticos positivos correlacionaram-se negativamente (p=0,005). Na DA, sintomas obsessivo-compulsivos (p=0,013) e sintomas psicóticos desorganizados (p=0,009) correlacionaram-se negativamente com o teste de Faux-Pas, ao passo que sintomas maníacos correlacionaram-se negativamente com o FERT (p=0,033).

**Conclusão:**

Pacientes com DFTvc exibiram mais sintomas esquizofrênicos e maníacos do que aqueles com DA, e os SNP impactaram a cognição social de maneira diferente em cada condição.

## INTRODUCTION

 Frontotemporal dementia (FTD) is a clinical syndrome characterized by a progressive decline in personality, social behaviour, and cognitive functions, associated with an atrophy of the frontal and temporal lobes1. The behavioural variant (bvFTD) is the most common phenotype, characterized by behavioural disorders such as apathy, loss of empathy, altered eating habits, disinhibition, and stereotypical behaviours.^1,2^ Cognitive deficits, particularly in social cognition, such as Theory of Mind (ToM) and emotion recognition, are also common in bvFTD, which impacts social interaction.^3,4^

 Diagnosing bvFTD is challenging, particularly early on, due to its similarity to primary psychiatric disorders and other neurodegenerative conditions like Alzheimer’s disease (AD).^5,6^ Accurately distinguishing AD from bvFTD is essential to prevent mismanagement and to reduce health risks associated with incorrect diagnosis.^6^ Common neuropsychiatric symptoms (NPS), including depression, apathy, and anxiety, often overlap in dementias, leading to misdiagnosis.^7 ,8^ Apathy is highly prevalent in both AD and bvFTD.^9^ NPS are associated with faster disease progression and higher healthcare costs.^5,10^ It is well established that social cognition deficits are present in bvFTD, due to fronto-striatal involvement, but the precise impact of NPS on its social cognition remains unclear. While the impact of apathy on social cognition has been studied^3^ , the effects of other NPS are less explored. 

 Given the high prevalence of NPS and their implications for differential diagnosis between bvFTD and AD, this study aimed to investigate NPS in bvFTD, in comparison to AD, and to explore correlations between these symptoms and social cognition. We posited that the influence of NPS on social cognition would manifest distinctively in individuals with bvFTD compared to those with AD, likely due to the differential topographical brain alterations associated with each condition^11^ . 

## METHODS

 Participants were recruited from the Cognitive Neurology Outpatient Clinic at the Hospital das Clínicas, UFMG, Brazil. The study included 13 patients with probable bvFTD, according to consensual diagnostic criteria2, and 18 patients with typical AD^12^. Groups were matched by sex, educational level, and dementia severity (see Methods in Supplementary Material — available at  https://www.demneuropsy.org/wp-content/uploads/2026/01/DN-2025.0409-Supplementary-Material.docx). Exclusion criteria included severe neurological or psychiatric disorders (e.g., previous stroke, epilepsy, schizophrenia, bipolar disorder), relevant medical comorbidities affecting cognition, marked cerebrovascular disease (Fazekas scale ≥2), atypical AD presentations, and bvFTD with primary progressive aphasia. The study was approved by the local ethics committee, and all participants provided written informed consent prior to participation. 

 Neuropsychiatric assessment included the Montgomery & Asberg Depression Rating Scale (MADRS)^13^, the Hamilton Anxiety Scale (HAM-A)^14^ , the Young Mania Rating Scale (YMRS)^15^ , the Revised Obsessive-Compulsive Inventory (OCI-R)^16^ , and the Starkstein Apathy Scale^17^ . The Positive and Negative Syndrome Scale (PANSS) was used to assess schizophrenic spectrum symptoms^18^ . The analysis utilized both subscales: Positive (PANSS-P), Negative (PANSS-N), and General symptoms (PANSS-G), as well as a five-factor model encompassing positive, negative, disorganization/cognition, affective, and resistance/hostility factors (see Methods in Supplementary Material). For all these above-mentioned neuropsychiatric instruments, higher scores indicate greater symptom severity. 

 All participants completed the Clinical Dementia Rating (CDR) assessment^19^ . Patients also underwent neuropsychological assessment (see Methods in Supplementary Material). Social cognition was evaluated with the Mini-Social and Emotion Assessment (mini-SEA), comprising a shortened *Faux-Pas* test (ToM) and the Facial Emotion Recognition Test (FERT)^3^ . A higher MiniSEA score indicates better social cognition performance. 

 Statistical analyses were performed with Statistical Package for the Social Sciences (SPSS) 22.0. Non-parametric tests were used due to non-normal data distribution: Mann-Whitney for group comparisons and Spearman’s test for correlations. Effect size (r) and Common Language Effect Size (CLES) were calculated. Significance was set at α=0.05 (see Methods in Supplementary Material). 

## RESULTS

 As shown in Table 1, bvFTD and AD groups did not differ significantly in sex, education, age at onset, symptoms’ duration, or CDR, although AD patients were older (p=0.034). 

**Table 1:** Neuropsychiatric symptoms in the Alzheimer’s disease and behavioural frontotemporal dementia groups.

		AD n=18	bvFTD n=13	p-value^*^	r^†^	CLES^‡^
Demographic						
	Male sex ‘n’ (%)	6 (33.3)	7 (53.8)	0.253^§^	-	-
	Age	71.5 (67.0–79.5)	66.0 (62.5–70.0)	0.034^//^	0.37	72.4%^¶^
	Education (years)	11.0 (4.0–16.0)	11.0 (4.0–16.0)	0.594	0.10	56.0%^¶^
	Age of onset	68.0 (60.0–74.3)	62.0 (56.0–67.5)	0.211	0.23	63.7%^¶^
	Duration of illness (years)	4.0 (2.8–6.5)	4.0 (2.0–5.0)	0.332	0.17	60.5%^¶^
	CDR	1.0 (0.5–1.0)	1.0 (0.5–1.0)	0.772	0.05	52.6%^¶^
Neuropsychiatric symptoms						
	MADRS	13.5 (9.0–16.5)	14.0 (6.5–24.5)	0.828	0.04	52.4%^#^
	HAM-A	12.5 (8.0–19.3)	14.0 (9.0–19.0)	0.540^//^	0.12	56.8%%^#^
	HAM-A-Psi	9.5 (6.8–12.3)	9.0 (6.5–14.5)	0.650	0.09	54.9%^#^
	HAM-A-Som	4.0 (1.8–6.0)	3.0 (2.0–8.0)	0.798	0.05	53.0%^b^
	YMRS	1.0 (0.0–3.0)	5.0 (3.0–8.0)	<0.001^**^	0.68	90.4%^#^
	OCI-R	10.0 (1.8–16.3)	15.0 (7.0–23.0)	0.089	0.31	68.2%^#^
	Checking	1.5 (0.0–3.3)	2.0 (0.0–4.5)	0.890	0.03	51.5%^#^
	Hoarding	1.0 (0.0–4.5)	4.0 (2.5–6.0)	0.106	0.30	67.5%^#^
	Neutralizing	0.0 (0.0–0.0)	0.0 (0.0–1.0)	0.258	0.21	62.2%^#^
	Obsession	0.0 (0.0–2.0)	1.0 (0.0–4.0)	0.146	0.27	65.6%^#^
	Ordering	1.5 (0.0–6.0)	6.0 (2.5–6.5)	0.028^//^	0.40	73.2%^#^
	Washing	0.0 (0.0–1.0)	1.0 (0.0–3.0)	0.170	0.25	64.7%^#^
	PANSS-Total	50.0 (44.8–54.8)	69.0 (57.0–72.0)	<0.001^**^	0.73	93.2%^#^
	PANSS-P Total	8.0 (7.0–9.3)	9.0 (8.5–11.5)	0.031^//^	0.39	73.1%^#^
	PANSS-N Total	14.0 (11.8–16.0)	20.0 (16.5–25.5)	<0.001^**^	0.72	92.7%^#^
	PANSS-G Total	29.0 (25.0–32.3)	36.0 (31.5–40.0)	<0.001^**^	0.67	89.3%^#^
	Positive symptoms^††^	4.0 (4.0–4.0)	4.0 (4.0–6.0)	0.075	0.33	69.2%^#^
	Negative symptoms^‡‡^	11.5 (9.0–13.3)	17.0 (12.5–22.5)	<0.001^**^	0.58	84.0%^#^
	Disorganization/Cognitive^§§^	9.0 (7.0–11.3)	10.0 (8.0–14.0)	0.373	0.17	59.6%^#^
	Affect/Depression-Anxiety^////^	7.5 (6.8–9.0)	8.0 (7.0–9.5)	0.540	0.12	56.6%^#^
	Resistance/Activation^¶¶5^	5.0 (4.0–8.0)	8.0 (6.0–10.5)	0.034^//^	0.38	72.6%^#^
	Apathy Scale	22.5 (14.8–29.0)	28.0 (21.0–34.5)	0.097	0.30	67.7%^#^
	CBI-R	64.5 (39.8–75.8)	66.0 (45.5–79.5)	0.737	0.06	53.6%^#^

Abbreviations, AD, Alzheimer’s Disease; bvFTD, Behavioral Variant Frontotemporal Dementia; CDR, Clinical Dementia Rating; MMSE, Mini-Mental State Examination, MADRS, Montgomery & Asberg Depression Rating Scale, HAM-A, Hamilton Anxiety Rating Scale, HAM-A Psi., Psychic Anxiety Subscale, HAM-A Som., Somatic Anxiety Subscale, YMRS, Young Mania Rating Scale, OCI-R, Obsessive-Compulsive Inventory-Revised, PANSS, Positive and Negative Syndrome Scale, PANSS P Total, Positive Subscale, PANSS N Total, Negative Subscale, PANSS G Total, General Psychopathology Subscale, CBI-R, Cambridge Behavioral Inventory-Revised.Notes: The values are represented in median and interquartile ranges (Q1–Q3).

* Mann-Whitney Test;

† ‘r’: Effect size (r) calculated based on the Mann-Whitney test statistics between AD and bvFTD groups: r<0.1 (negligible); 0.1≤r<0.3 (small); 0.3≤r<0.5 (medium); r≥0.5 (large);

‡ Common Language Effect Size (CLES): calculated based on the Mann-Whitney test statistics between bvFTD and AD;

§ χ^2^ Test;

// p<0.05;

¶ The value corresponds to the probability of a randomly selected person from the AD group having a higher score than a randomly selected person from the bvFTD group;

# The value corresponds to the probability of a randomly selected person from the bvFTD group having a higher score than a randomly selected person from the AD group;

** p<0.001;

†† Positive Symptoms Factor: PANSS P1, P3, P6, and G9;

‡‡ Negative Symptoms Factor: PANSS N1, N2, N3, N4, N6, G7, and G16;

§§ Disorganization/Cognition Factor: PANSS P2, N5, N7, G11, and G13;

//// Affective (Anxiety-Depression) Factor: PANSS G2, G3, and G6;

¶¶ Resistance or Hostility/Activation: PANSS P4, P7, G8, and G14

 Among the participants, two patients from the bvFTD group and six patients from the AD group were unable to complete the social cognition tests (Mini-SEA, Faux-Pas, FERT) due to difficulties with comprehension or engagement. There were no significant differences in the neuropsychological performance (global cognitive efficiency and episodic memory) or in the social cognition tests (*Faux-Pas* and FERT) between the groups (Supplementary Material Table S1). 

 Regarding NPS (Table 1), there was no difference in MADRS, HAM-A, and apathy scores between AD and bvFTD groups. Total OCI-R scores also did not differ; however, bvFTD patients scored significantly higher on the "ordering" subscale (p=0.028). bvFTD patients exhibited significantly higher manic symptom scores on the YMRS compared to AD patients (p<0.001). The CLES analysis indicates a 90.4% probability that a bvFTD patient will have higher mania scores than an AD patient. The groups also differed significantly on the PANSS-total scale (p<0.001), with bvFTD patients having a higher total score (93.2% probability in CLES). Differences were found across all three PANSS subscales, with bvFTD scoring higher in PANSS-P (p=0.031), PANSS-N (p<0.001), and PANSS-G (p<0.001) (Table 1 and Supplementary Table S2). PANSS five-factor model analysis revealed higher scores in bvFTD for "Negative Symptoms" (p<0.001) and "Resistance Symptoms" (p=0.034) factors, but no difference in the "Positive Symptoms" factor. 

**Figure 1:**
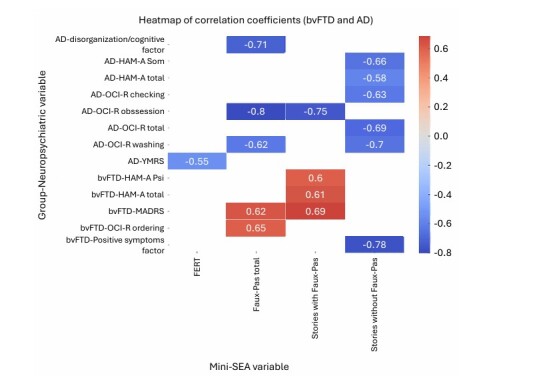
Heatmap with the significant Spearman correlations between neuropsychiatric symptoms and social cognition (Mini-Social and Emotional Assessment) in behavioural frontotemporal dementia and Alzheimer’s disease patients. The numbers inside the heatmap boxes indicate the Spearman correlation coefficient. Significance was set at α=0.05.

Abbreviations: AD, Alzheimer’s disease; bvFTD, behavioural frontotemporal dementia; HAM-A, Hamilton Anxiety Rating Scale; HAM-A Psi., Psychic Anxiety Subscale; HAM-A Som., Somatic Anxiety Subscale; MADRS, Montgomery & Asberg Depression Rating Scale; MiniSEA, Mini-Social and Emotional Assessment; OCI-R, Obsessive-Compulsive Inventory-Revised.Notes: Positive Symptoms Factor: PANSS P1, P3, P6, and G9. Disorganization/Cognition Factor: PANSS P2, N5, N7, G11, and G13.

 Spearman’s test revealed significant correlations between NPS and social cognition, with different patterns in bvFTD and AD (Figure 1 and Supplementary Table S3). In bvFTD, the "Positive Symptoms" from the PANSS five-factor model strongly and negatively correlated with the *Faux-Pas* test (Rho=-0.778, p=0.005). Depressive (MADRS) and anxious symptoms (HAM-A) showed moderate positive correlations (Rho=0.020 to 0.049, p<0.05) with the Faux-Pas test. OCI-R Ordering also showed a moderate positive correlation with the *Faux-Pas* test (Rho=0.649, p=0.031). 

 Conversely, in AD patients (Figure 1 and Supplementary Table S3), anxious symptoms (HAM-A), manic symptoms (YMRS), obsessive-compulsive symptoms — OCS (OCI-R Total, along with the following subscales: Checking, Obsession, and Washing), and the "Disorganization/Cognition" from the PANSS five-factor model showed moderate to strong negative correlations with performance on social cognition tests of the Mini-SEA. 

## DISCUSSION

 This exploratory study examines the interface between NPS and social cognition in bvFTD and AD. Our neuropsychiatric evaluation revealed both similarities and differences in NPS between the two conditions. We also found that NPS impact social cognition test performance in bvFTD and AD patients, but in different ways. 

 Consistent with previous studies^20,21^, we found no significant differences in depression and anxiety scores between bvFTD and AD groups, confirming the prevalence of these symptoms in both. This highlights the clinical relevance of screening and managing these symptoms in both conditions. 

 bvFTD patients showed greater OCI-R scores for ‘ordering’ symptoms than AD, aligning with previous findings that show up to 82% of bvFTD patients exhibit OCS like checking and hoarding^22^. These OCS, including ordering, directly meet the diagnostic criteria ‘D’ for bvFTD^2^. Our findings suggest the OCI-R might be useful in detecting OCS in bvFTD patients. 

 Manic-like symptoms, measured by YMRS, were significantly higher in bvFTD than AD, in line with prior data^23^. The similarity of symptoms between bvFTD and primary mania, such as euphoria, disinhibition, and impulsivity, may explain the greater YMRS scores in bvFTD^24^. Disinhibition in bvFTD leads to inappropriate and impulsive behaviors without awareness of social boundaries^1^. Patients with bvFTD, unlike those with bipolar disorder, do not show remorse due to anosognosia^5^. 

 The PANSS assesses schizophrenia-related symptoms across Positive Symptoms, Negative Symptoms, and General Psychopathology subscales^18^. Compared to the AD group, bvFTD patients showed dominance of negative symptoms, such as blunted affect, emotional withdrawal, poor rapport, passive social withdrawal, and lack of conversational spontaneity. The symptoms described align with studies on bvFTD^25^ and the diagnostic criteria established by Rascovsky^2^: emotional/social withdrawal corresponding to diminished responsiveness (C.1) and loss of social interest (C.2), while blunted affect and poor rapport exemplify apathy (B.1) and inertia (B.2). 

 While the traditional PANSS Positive subscale (PANSS-P) differentiated bvFTD and AD groups, the PANSS five-factor model’s “Positive Symptoms Factor”, which excludes excitation (P4) and hostility (P7) but includes delusions (P1) and hallucinations (P3), showed no statistical differences between groups. Conversely, the bvFTD group exhibited significant elevation in the “Resistance Factor”, encompassing ‘excitation’ (P4), ‘hostility’ (P7), ‘uncooperativeness’ (G8), and ‘poor impulse control’ (G14). These items of “Resistance Factor” align strongly with Rascovsky’s^2^ Criterion A: early behavioral disinhibition (A), including socially inappropriate behaviour (A.1), loss of manners (A.2), and impulsive actions (A.3), providing a distinct neuropsychiatric hallmark for bvFTD. 

 Strong negative correlations were observed between psychotic symptoms and social cognition in bvFTD and AD. Specifically, the PANSS "Positive Symptoms" factor in bvFTD and the "Disorganization/Cognition" factor in AD were associated with poorer *Faux-Pas* test performance, indicating greater difficulty interpreting social and emotional cues. These findings align with evidence linking ToM deficits in schizophrenia to psychotic symptom severity^26,27^. 

 In bvFTD, a paradoxical positive correlation emerged between depressive/anxious symptoms and Faux-Pas test performance, suggesting that heightened negative emotional states and anxiety might enhance attention to social and emotional details, potentially improving perspective-taking^28,29^. Conversely, AD patients showed a negative correlation between anxiety and social cognition, underscoring disease-specific effects of anxiety on social abilities. 

 Only the AD group showed a correlation between YMRS and social cognition performance. Research on the impact of manic symptoms on social cognition in bvFTD and AD is limited. However, these findings align with previous studies in bipolar disorder, which indicate that manic symptoms impair social cognition^30^. Further research with larger samples is necessary to systematically explore these relationships. 

 The similar scores in social cognition task between bvFTD and AD is an unexpected finding, but may be due to the small sample size. Notwithstanding this caveat, the distinct NPS-social cognition correlations between bvFTD and AD groups reflect different, complex interactions between divergent neuropathological, topographical involvement and psychiatric profiles across both disorders, with unique neuropsychiatric profiles modulating social dysfunction via divergent neural substrates. Unfortunately, the absence of neuroimaging data hampers the investigation of these potential interactions^4,11^. 

 Our study has limitations, including small sample size, which may account for the lack of significant differences on cognitive tests. The absence of genetic information, and pharmacological treatment factors hinders our analysis. Crucially, the absence of neuroimaging data prevented correlation with lesion topography and cognitive/neuropsychiatric findings. We also did not apply Bonferroni correction due to the sample size and the exploratory nature of the research. Given the small sample size, we did not apply linear regression or Generalized Linear Models. Future studies should consider multivariate analysis in the investigation of interaction between neuropsychiatric and social cognition measures. Being cross-sectional, this study cannot capture the dynamic interactions between NPS and cognitive changes over time, highlighting the need for longitudinal studies. 

 This research provides a comparative analysis of NPS in bvFTD and AD, highlighting the connections between these NPS and social cognition in dementia. Further studies are needed to explore these findings and their implications for diagnosis and management. 

## Data Availability

The datasets generated and/or analyzed during the current study are not publicly available due to [ethical/legal/privacy] restrictions but are available from the corresponding author upon reasonable request.

## References

[1] Boeve BF, Boxer AL, Kumfor F, Pijnenburg V, Rohrer JD (2022). Advances and controversies in frontotemporal dementia: diagnosis, biomarkers, and therapeutic considerations. Lancet Neurol.

[2] Rascovsky K, Hodges JR, Knopman D, Mendez MF, Kramer JH, Neuhaus J (2011). Sensitivity of revised diagnostic criteria for the behavioural variant of frontotemporal dementia. Brain.

[3] Mariano LI, Caramelli P, Guimarães HC, Gambogi LB, Moura MVB, Yassuda MS (2020). Can social cognition measurements differentiate behavioral variant frontotemporal dementia from Alzheimer’s disease regardless of apathy?. J Alzheimers Dis.

[4] Bertoux M, Volle E, Funkiewiez A, Souza LC, Leclercq D, Dubois B (2012). Social Cognition and Emotional Assessment (SEA) is a marker of medial and orbital frontal functions: a voxel-based morphometry study in behavioral variant of frontotemporal degeneration. J Int Neuropsychol Soc.

[5] Ducharme S, Pearl-Dowler L, Gossink F, McCarthy J, Lai J, Dickerson BC (2019). The Frontotemporal Dementia versus Primary Psychiatric Disorder (FTD versus PPD) checklist: a bedside clinical tool to identify behavioral variant FTD in patients with late-onset behavioral changes. J Alzheimers Dis.

[6] Souza LC, Brucki SMD, Schilling LP, Silva LC, Takada LT, Bahia VS (2023). Current clinical and research practices on frontotemporal dementia in Brazil: a national survey. Arq Neuropsiquiatr.

[7] Brodaty H, Connors MH, Xu J, Woodward M, Ames D, PRIME study group (2015). The course of neuropsychiatric symptoms in dementia: a 3-year longitudinal study. J Am Med Dir Assoc.

[8] Zhao QF, Tan L, Wang HF, Jiang T, Tan MS, Tan L (2016). The prevalence of neuropsychiatric symptoms in Alzheimer’s disease: systematic review and meta-analysis. J Affect Disord.

[9] Wei G, Irish M, Hodges JR, Piguet O, Kumfor F (2020). Disease-specific profiles of apathy in Alzheimer’s disease and behavioural-variant frontotemporal dementia differ across the disease course. J Neurol.

[10] Da Silva TBL, Ordonez TN, Bregola AG, Bahia VS, Cecchini MA, Guimarães HC (2021). Neuropsychiatric symptoms in behavioral variant frontotemporal dementia and Alzheimer’s disease: a 12-month follow-up study. Front Neurol.

[11] Ramanan S, Souza LC, Moreau N, Sarazin M, Teixeira AL, Allen Z (2017). Determinants of theory of mind performance in Alzheimer’s disease: a data-mining study. Cortex.

[12] McKhann GM, Knopman DS, Chertkow H, Hyman BT, Jack CR, Kawas CH (2011). The diagnosis of dementia due to Alzheimer’s disease: recommendations from the National Institute on Aging-Alzheimer’s Association workgroups on diagnostic guidelines for Alzheimer’s disease. Alzheimers Dement.

[13] Montgomery SA, Asberg M (1979). A new depression scale designed to be sensitive to change. Br J Psychiatry.

[14] Hamilton M (1959). The assessment of anxiety states by rating. Br J Med Psychol.

[15] Young RC, Biggs JT, Ziegler VE, Meyer DA (1978). A rating scale for mania: reliability, validity and sensitivity. Br J Psychiatry.

[16] Foa EB, Huppert JD, Leiberg S, Langner R, Kichic R, Hajcak G (2002). The obsessive-compulsive inventory: development and validation of a short version. Psychol Assess.

[17] Starkstein SE, Mayberg HS, Preziosi TJ, Andrezejewski P, Leiguarda R, Robinson RG (1992). Reliability, validity, and clinical correlates of apathy in Parkinson’s disease. J Neuropsychiatry Clin Neurosci.

[18] Shafer A, Dazzi F (2019). Meta-analysis of the positive and Negative Syndrome Scale (PANSS) factor structure. J Psychiatr Res.

[19] Morris JC (1993). The Clinical Dementia Rating (CDR): current version and scoring rules. Neurology.

[20] Liu S, Liu J, Wang XD, Shi Z, Zhou Y, Li J (2018). Caregiver burden, sleep quality, depression, and anxiety in dementia caregivers: a comparison of frontotemporal lobar degeneration, dementia with Lewy bodies, and Alzheimer’s disease. Int Psychogeriatr.

[21] Chakrabarty T, Sepehry AA, Jacova C, Hsiung GYR (2015). The prevalence of depressive symptoms in frontotemporal dementia: a meta-analysis. Dement Geriatr Cogn Disord.

[22] Perry DC, Whitwell JL, Boeve BF, Pankratz VS, Knopman DS, Petersen RC (2012). Voxel-based morphometry in patients with obsessive-compulsive behaviors in behavioral variant frontotemporal dementia. Eur J Neurol.

[23] Cajanus A, Solje E, Koikkalainen J, Lötjönen J, Suhonen NM, Hallikainen I (2019). The Association Between Distinct Frontal Brain Volumes and Behavioral Symptoms in Mild Cognitive Impairment, Alzheimer’s Disease, and Frontotemporal Dementia. Front Neurol.

[24] Woolley JD, Khan BK, Murthy NK, Miller BL, Rankin KP (2011). The diagnostic challenge of psychiatric symptoms in neurodegenerative disease; rates of and risk factors for prior psychiatric diagnosis in patients with early neurodegenerative disease. J Clin Psychiatry.

[25] Gossink FT, Vijverberg EG, Krudop W, Scheltens P, Stek ML, Pijnenburg YA (2017). Psychosis in behavioral variant frontotemporal dementia. Neuropsychiatr Dis Treat.

[26] Cruz BF, Oliveira AM, Del-Ben CM, Corcoran R, Salgado JV (2022). Validation of the Brazilian version of the Hinting Task and Facial Emotion Recognition Test (FERT-100) in patients with schizophrenia. Dement Neuropsychol.

[27] Negrão JG, Osório AAC, Bressan R, Gadelha A, Lederman VRG, Tafla TL (2023). Social cognition in individuals with schizophrenia, autism spectrum disorder and controls. J Bras Psiquiatr.

[28] Weightman MJ, Air TM, Baune BT (2014). A review of the role of social cognition in major depressive disorder. Front Psychiatry.

[29] Zainal NH, Newman MG (2018). Worry amplifies theory-of-mind reasoning for negatively valenced social stimuli in generalized anxiety disorder. J Affect Disord.

[30] Barbosa IG, Leite FMC, Bertoux M, Guimarães HC, Mariano LI, Gambogi LB (2023). Social cognition across bipolar disorder and behavioral variant frontotemporal dementia: an exploratory study. Braz J Psychiatry.

