# Supplementary material for: Neuropsychiatric symptoms and social cognition in frontotemporal dementia and Alzheimer’s disease
Source: Dement Neuropsychol. 2026 Jun 15;20:e20250409. doi: 10.1590/1980-5764-DN-2025-0409 (PMC13268645; doi:10.1590/1980-5764-DN-2025-0409)
Supplement: Supplementary Materials [file 1980-5764-dn-20-e20250409-md1.docx]

**Supplementary Material 1**

**Methods**

*Dementia stages*

Patients from both groups were at mild to moderate stages of dementia, as assessed by the Frontotemporal Dementia Rating Scale^1,2^ and the Clinical Dementia Rating (CDR)^3,4^ for bvFTD and AD patients, respectively.

*Neuropsychiatric Assessment*

We applied the Positive and Negative Syndrome Scale (PANSS) for schizophrenic spectrum symptoms^5^. Of note, we considered both the traditional subscales division (PANSS P - Positive Symptoms; PANSS N - Negative Symptoms and PANSS G - General Symptoms) and the five-factor model to analyse the scores provided by the PANSS^6^, as follows: 1º) Positive Symptoms Factor: PANSS P1, P3, P6, and G9; 2º) Negative Symptoms Factor: PANSS N1, N2, N3, N4, N6, G7, and G16; 3º) Disorganization/Cognition Factor: PANSS P2, N5, N7, G11, and G13; 4º) Affective (Anxiety-Depression) Factor: PANSS G2, G3, and G6; 5º) Resistance or Hostility/Activation: PANSS P4, P7, G8, and G14.

*Neuropsychological Assessment*

Patients underwent a comprehensive cognitive evaluation with the following tools: the Mini-Mental State Examination (MMSE)^7,8^, Addenbrooke’s Cognitive Examination Revised (ACE-R)^9,10^, Frontal Assessment Battery (FAB)^11,12^, Hayling Test^13,14^, WAIS-III Digit Span^15^, and Verbal Fluency Test (animal category and phonological test “F,” “A,” and “S”)^16,17^. The short version of the Social and Emotion Assessment (Mini-SEA) was used for social cognition, comprising a shortened version of the Faux-Pas test to assess Theory of Mind (ToM) and the facial emotion recognition test (FERT).^18,19^

*Statistical Analyses*

The Shapiro-Wilk test showed a non-normal distribution, prompting the use of non-parametric methods. The strength of Spearman's correlations coefficients was determined according to established statistical benchmarks^20^. Effect size (r) was calculated from the Mann-Whitney test's Ζ statistic. Additionally, the Common Language Effect Size (CLES), based on the Mann-Whitney U test between bvFTD and AD, was used to estimate the probability that a randomly selected individual from the target group would have a higher score than one from the comparison group^21^.

**Supplementary Table** 1: Cognitive profile of the Alzheimer's disease (AD) and behavioural variant frontotemporal dementia (bvFTD) groups.

|  | **AD**  **n=18** | **bvFTD**  **n=13** | **p value**^†^ | **r^§^** | **CLES**^‡^ |
| --- | --- | --- | --- | --- | --- |
| **MMSE** | 24,0 (20,0 – 25,3) | 25,0 (22,0 – 28,5) | 0,170 | 0,25 | 64,7%^b^ |
| **ACE-R** | 63,0 (47,0-73,0) | 70,0 (50,0 – 76,0) | 0,525 | 0,13 | 57,4%^b^ |
| **Span Direct**  **Span Inverse** | 4,0 (4,0 – 5,0)  3,0 (2,0 – 4,0) | 5,0 (3,0 – 5,0)  3,0 (2,0 – 4,5) | 0,964  0,618 | 0,00  0,10 | 50,5%^c^  55,6%^b^ |
| **Hayling part B (Erros/15´)**  **Hayling part B (Erros/45´)**  **Hayling part B - A** | 9,0 (7,0 – 11,0)  15,0 (9,0 – 21,0)  35,7 (20,1 – 118,6) | 6,0 (2,3 – 11,5)  9,5 (3,3 – 19,5)  40,7 (21,4 – 56,3) | 0,139  0,152  0,755 | 0,29  0,28  0,06 | 67,2%^c^  66,7%^c^  53,9%^c^ |
| **FAB** | 11,0 (8,0 – 15,0) | 14,0 (9,5 – 15,5) | 0,294 | 0,20 | 61,8%^b^ |
| **Animal Fluency** | 9,0 (5,0 – 13,0) | 8,0 (4,5 – 12,5) | 0,496 | 0,13 | 57,9%^c^ |
| **Verbal fluency - Total (FAS)** | 30,0 (18,0 – 38,0) | 28,0 (10,0 – 40,0) | 0,496 | 0,13 | 57,9%^c^ |
| **Stories with *Faux-Pas*** | 18,0 (16,0 – 22,0) | 15,0 (14,0 – 24,0) | 0,316 | 0,21 | 62,5%^c^ |
| **Stories without *Faux-Pas*** | 10,0 (8,0 – 10,0) | 10,0 (8,0 – 10,0) | 0,525 | 0,13 | 58,0%^c^ |
| ***Faux-Pas* total** | 27,5 (24,5 – 32,0) | 25,0 (19,0 – 28,0) | 0,151 | 0,30 | 67,8%^c^ |
| ***Faux-Pas* Control** | 16,5 (15,0 – 18,0) | 19,0 (14,0 – 20,0) | 0,235 | 0,26 | 64,8%^b^ |
| **Ekman Total**  **Happiness**  **Surprise**  **Disgust**  **Fear**  **Anger**  **Sadness**  **Neutral** | 23,0 (19,0 – 27,0)  5,0 (5,0 – 5,0)  4,0 (2,0 – 5,0)  4,0 (3,0 – 5,0)  1,0 (0,0 – 1,0)  4,0 (2,0 – 5,0)  3,0 (2,0 – 4,0)  5,0 (3,0 – 5,0) | 18,0 (14,0 – 25,5)  5,0 (4,5 – 5,0)  3,0 (2,5 – 5,0)  1,0 (0,5 – 4,0)  1,0 (0,0 – 1,5)  3,0 (1,0 – 3,5)  3,0 (1,5 – 4,0)  4,0 (1,0 – 5,0) | 0,156  0,683  0,892  0,046^a^  0,892  0,156  0,786  0,294 | 0,27  0,07  0,03  0,37  0,03  0,27  0,05  0,20 | 65,9%^c^  54,6%^c^  51,5%^b^  72,1%^c^  51,8%^c^  66,2%^c^  53,3%^c^  62,1%^c^ |

The values are represented in median and interquartile ranges (Q1 – Q3).

^†^Mann-Whitney Test.

^*^p < 0.05; ^**^p < 0.01; ^***^p < 0.001.

^§^‘r’: Effect size (r) calculated based on the Mann-Whitney test statistics between AD and bvFTD groups: r < 0.1 (negligible); 0.1 ≤ r < 0.3 (small); 0.3 ≤ r < 0.5 (medium); r ≥ 0.5 (large).

^‡^Common Language Effect Size (CLES): calculated based on the Mann-Whitney test statistics between bvFTD and AD. ^b^The value corresponds to the probability of a randomly selected person from the bvFTD group having a higher score than a randomly selected person from the AD group. ^c^The value corresponds to the probability of a randomly selected person from the AD group having a higher score than a randomly selected person from the bvFTD group.

**ACE-R**: Addenbrooke's Cognitive Examination – Revised, **AD**: Alzheimer's Disease; **bvFTD**: Behavioral Variant Frontotemporal Dementia; **FAB**: Frontal Assessment Battery; **MMSE**: Mini-Mental State Examination; **Total Verbal Fluency (FAS)**: Words beginning with the letters F, A, and S.

**Supplementary Table 2**: PANSS scale broken down into all its items and subscales for the bvFTD and AD groups.

|  | **AD**  **n=18** | **bvFTD**  **n=13** | **p value**^†^ | **r^§^** | **CLES**^‡^ |
| --- | --- | --- | --- | --- | --- |
| P1 - Delusions | 1,0 (1,0 – 1,0) | 1,0 (1,0 – 1,5) | 0,417 | 0,15 | 58,8%^b^ |
| P2 – Conceptual disorganisation | 1,0 (1,0 – 2,3) | 2,0 (1,0 – 3,0) | 0,226 | 0,23 | 63,2%^b^ |
| P3 – Hallucinatory behaviour | 1,0 (1,0 – 1,0) | 1,0 (1,0 – 1,0) | 0,737 | 0,07 | 53,8%^b^ |
| P4 – Excitement | 1,0 (1,0 – 1,0) | 1,0 (1,0 -1,0) | 0,650 | 0,09 | 55,1%^b^ |
| P5 – Grandiosity | 1,0 (1,0 – 1,0) | 1,0 (1,0 – 1,0) | 0,489 | 0,13 | 57,7%^b^ |
| P6 – Suspiciousness/persecution | 1,0 (1,0 – 1,0) | 1,0 (1,0 – 1,0) | 0,489 | 0,13 | 57,7%^b^ |
| P7 – Hostility | 1,0 (1,0 – 2,3) | 2,0 (1,0 – 3,0) | 0,226 | 0,22 | 63,0%^b^ |
| **PANSS-P** | **8,0 (7,0 – 9,3)** | **9,0 (8,5 – 11,5)** | **0,031^*^** | **0,39** | **73,1%**^b^ |
| N1 – Blunted affect | 1,5 (1,0 – 2,0) | 3,0 (2,0 – 4,0) | <0,001^***^ | 0,65 | 88,5%^b^ |
| N2 – Emotional withdrawal | 3,0 (2,0 – 3,3) | 4,0 (2,5 – 5,0) | 0,031^*^ | 0,39 | 72,9%^b^ |
| N3 – Poor rapport | 1,0 (1,0 – 2,0) | 2,0 (2,0 – 4,0) | <0,001^***^ | 0,65 | 88,7%^b^ |
| N4 – Passive/apathetic social  withdrawal | 2,5 (1,8 – 3,0) | 4,0 (3,0 – 4,5) | 0,006^**^ | 0,49 | 78,8%^b^ |
| N5 – Difficulty in abstract thinking | 3,0 (2,0 – 3,0) | 4,0 (2,0 – 4,0) | 0,135 | 0,27 | 66,0%^b^ |
| N6 – Lack of spontaneity and  flow of conversation | 1,0 (1,0 – 1,0) | 2,0 (1,0 – 3,0) | 0,010 ^*^ | 0,46 | 77,4%^b^ |
| N7 – Stereotyped thinking | 1,5 (1,0 – 3,0) | 3,0 (1,0 – 3,5) | 0,373 | 0,17 | 59,6%^b^ |
| **PANSS-N** | **14,0 (11,8 – 16,0)** | **20,0 (16,5 – 25,5)** | **<0,001**^***^ | **0,72** | **92,7%**^b^ |
| G1 – Somatic concern | 1,0 (1,0 – 1,0) | 1,0 (1,0 - 3,0) | 0,157 | 0,26 | 65,4%^b^ |
| G2 – Anxiety | 3,0 (2,8 – 4,0) | 4,0 (3,0 – 5,0) | 0,042^*^ | 0,37 | 71,8%^b^ |
| G3 – Guilty feelings | 1,0 (1,0 – 1,0) | 1,0 (1,0 – 1,0) | 0,953 | 0,01 | 50,9%^c^ |
| G4 – Tension | 2,0 (1,0 – 3,0) | 3,0 (2,0 – 3,5) | 0,011^*^ | 0,46 | 76,9%^b^ |
| G5 – Mannerisms/posturing | 1,0 (1,0 – 1,0) | 1,0 (1,0 – 1,0) | 0,489 | 0,13 | 57,7%^b^ |
| G6 – Depression | 3,5 (3,0 – 4,0) | 3,0 (2,0 – 4,0) | 0,514 | 0,12 | 57,3%^c^ |
| G7 – Motor retardation | 1,0 (1,0 – 1,0) | 1,0 (1,0 – 3,0) | 0,068 | 0,33 | 69,7%^b^ |
| G8 – Uncooperativeness | 1,0 (1,0 – 2,0) | 3,0 (1,5 – 3,5) | 0,028^*^ | 0,40 | 73,5%^b^ |
| G9 – Unusual thought content | 1,0 (1,0 – 1,0) | 1,0 (1,0 – 1,0) | 0,489 | 0,13 | 57,7%^b^ |
| G10 – Desorientation | 3,0 (3,0 – 3,0) | 2,0 (1,5 – 3,0) | 0,125 | 0,27 | 66,5%^c^ |
| G11 – Poor attention | 2,0 (1,8 – 3,0) | 2,0 (1,0 – 3,0) | 0,767 | 0,06 | 53,4%^b^ |
| G12 – Lack of judgement & insight | 2,5 (1,0 – 3,0) | 3,0 (3,0 – 4,0) | 0,038^*^ | 0,38 | 72,2%^b^ |
| G13 – Disturbance of volition | 1,0 (1,0 – 1,0) | 1,0 (1,0 – 1,0) | 1,000 | 0,00 | 50,0% |
| G14 – Poor impulse control | 1,0 (1,0 – 3,0) | 3,0 (2,0 – 3,0) | 0,031^*^ | 0,39 | 72,9%^b^ |
| G15 – Preoccupation | 1,0 (1,0 – 2,0) | 3,0 (1,5 – 3,0) | 0,025^*^ | 0,40 | 73,7%^b^ |
| G16 – Active social avoidance | 1,0 (1,0 – 1,0) | 1,0 (1,0 – 2,0) | 0,708 | 0,08 | 54,3%^b^ |
| **PANSS-G** | **29,0 (25,0 – 32,3)** | **36,0 (31,5 – 40,0)** | **<0,001^***^** | **0,67** | **89,3%**^b^ |
| **PANSS Total** | **50,0 (44,8 – 54,8)** | **69,0 (57,0-72,0)** | **<0,001^***^** | **0,73** | **93,2%**^b^ |

The values are represented in median and interquartile ranges (Q1 – Q3).

^†^Mann-Whitney Test.

^*^p < 0.05; ^**^p < 0.01; ^***^p < 0.001.

^§^‘r’: Effect size (r) calculated based on the Mann-Whitney test statistics between AD and bvFTD groups: r < 0.1 (negligible); 0.1 ≤ r < 0.3 (small); 0.3 ≤ r < 0.5 (medium); r ≥ 0.5 (large).

^‡^Common Language Effect Size (CLES): calculated based on the Mann-Whitney test statistics between bvFTD and AD. ^b^The value corresponds to the probability of a randomly selected person from the bvFTD group having a higher score than a randomly selected person from the AD group. ^c^The value corresponds to the probability of a randomly selected person from the AD group having a higher score than a randomly selected person from the bvFTD group.

**AD**: Alzheimer's Disease; **bvFTD**: Behavioral Variant Frontotemporal Dementia; **PANSS**: Positive and Negative Syndrome Scale, **PANSS P Total**: Positive Subscale, **PANSS N Total**: Negative Subscale, **PANSS G Total**: General Psychopathology Subscale.

**Supplementary Table 3:** Significant Correlations Between NPS and Social Cognition (Mini-SEA) in bvFTD and AD patients.

| **Neuropsychiatric Variable** | **Mini-SEA Variable** | **Correlation Coefficient (Rho)** ^†^ | **p Value** | **Strength of Correlation** |
| --- | --- | --- | --- | --- |
| **bvFTD** | | | | |
| MADRS | Stories with *Faux-Pas* | 0.687 | 0.020^*^ | Moderate |
|  | Faux-Pas Total | 0.624 | 0.040^*^ | Moderate |
| HAM-A-Psi | Stories with *Faux-Pas* | 0.604 | 0.049^*^ | Moderate |
| HAM-A Total | Stories with Faux-Pas | 0.614 | 0.045^*^ | Moderate |
| OCI-R Ordering | *Faux-Pas* Total | 0.649 | 0.031^*^ | Moderate |
| Positive symptoms factor^1^ | Stories without *Faux-Pas* | -0.778 | 0.005^**^ | Strong |
| **AD** | | | | |
| HAM-A Som | Stories without *Faux-Pas* | -0.655 | 0.021^*^ | Moderate |
| HAM-A total | Stories without *Faux-Pas* | -0.583 | 0.047^*^ | Moderate |
| YMRS | FERT | -0.551 | 0.033^*^ | Moderate |
| OCI-R Total | Stories without *Faux-Pas* | -0.688 | 0.013^*^ | Moderate |
| OCI-R Checking | Stories without *Faux-Pas* | -0.628 | 0.029^*^ | Moderate |
| OCI-R Obsession | Stories with *Faux-Pas* | -0.752 | 0.005^**^ | Strong |
|  | *Faux-Pas* Total | -0.803 | 0.002^**^ | Strong |
| OCI-R Washing | Stories without Faux-Pas | -0.697 | 0.012^*^ | Moderate |
|  | *Faux-Pas* Total | -0.625 | 0.030^*^ | Moderate |
| Disorganization/ Cognitive Factor^2^ | *Faux-Pas* Total | -0.712 | 0.009^**^ | Strong |

^†^(ρ) indicates the Spearman correlation coefficient.

^*^p < 0.05; ^**^p < 0.01; ^***^p < 0.001.

**HAM-A**: Hamilton Anxiety Rating Scale; **HAM-A Psi**.: Psychic Anxiety Subscale; **HAM-A Som**.: Somatics Anxiety Subscale; **MADRS**: Montgomery & Asberg Depression Rating Scale; **OCI-R**: Obsessive-Compulsive Inventory-Revised;

^1^**Positive** **Symptoms Factor**: PANSS P1, P3, P6, and G9.

^2^**Disorganization**/**Cognition Factor:** PANSS P2, N5, N7, G11, and G13.

**References**

1. Mioshi E, Hsieh S, Savage S, Hornberger M, Hodges JR. Clinical staging and disease progression in frontotemporal dementia. Neurology. 2010 May 18;74(20):1591–7.

2. Lima-Silva TB, Bahia VS, Cecchini MA, Cassimiro L, Guimarães HC, Gambogi LB, et al. Validity and Reliability of the Frontotemporal Dementia Rating Scale (FTD-FRS) for the Progression and Staging of Dementia in Brazilian Patients. Alzheimer Dis Assoc Disord. 2018;32(3):220–5.

3. Macedo Montaño MBM, Ramos LR. Validity of the Portuguese version of Clinical Dementia Rating. Rev Saúde Pública. 2005 Dec;39:912–7.

4. Morris JC. The Clinical Dementia Rating (CDR): current version and scoring rules. Neurology. 1993 Nov;43(11):2412–4.

5. Kay SR, Fiszbein A, Opler LA. The positive and negative syndrome scale (PANSS) for schizophrenia. Schizophr Bull. 1987;13(2):261–76.

6. Shafer A, Dazzi F. Meta-analysis of the positive and Negative Syndrome Scale (PANSS) factor structure. J Psychiatr Res. 2019 Aug;115:113–20.

7. Folstein MF, Folstein SE, McHugh PR. “Mini-mental state”. A practical method for grading the cognitive state of patients for the clinician. J Psychiatr Res. 1975 Nov;12(3):189–98.

8. Brucki SMD, Nitrini R, Caramelli P, Bertolucci PHF, Okamoto IH. Sugestões para o uso do mini-exame do estado mental no Brasil. Arq Neuro-Psiquiatr. 2003 Sep;61:777–81.

9. Mioshi E, Dawson K, Mitchell J, Arnold R, Hodges JR. The Addenbrooke’s Cognitive Examination Revised (ACE-R): a brief cognitive test battery for dementia screening. Int J Geriatr Psychiatry. 2006 Nov;21(11):1078–85.

10. Amaral-Carvalho V, Bento Lima-Silva T, Inácio Mariano L, de Souza LC, Cerqueira Guimarães H, Santoro Bahia V, et al. Predicting Dementia Due to Alzheimer’s Disease and Behavioral Variant Frontotemporal Dementia Using Algorithms with the Addenbrooke’s Cognitive Examination-Revised Subscores Combined with Sociodemographic Factors. Current Alzheimer Research. 2023 May 1;20(5):341–9.

11. Dubois B, Slachevsky A, Litvan I, Pillon B. The FAB: a Frontal Assessment Battery at bedside. Neurology. 2000 Dec 12;55(11):1621–6.

12. Beato RG, Nitrini R, Formigoni AP, Caramelli P. Brazilian version of the Frontal Assessment Battery (FAB): Preliminary data on administration to healthy elderly. Dement neuropsychol. 2007 Mar;1:59–65.

13. Burgess PW, Shallice T. The Hayling and Brixton tests. Bury St Edmunds: Thames Valley Test Company; 1997. 20 p.

14. Siqueira L de S, Scherer LC, Reppold CT, Fonseca RP. Hayling Test - adult version: applicability in the assessment of executive functions in children. Psychol Neurosci. 2010 Dec;3:189–94.

15. Wechsler D. WMS-III: Wechsler Memory Scale Administration and Scoring Manual. Psychological Corporation; 1997. 212 p.

16. Brucki SMD, Rocha MSG. Category fluency test: effects of age, gender and education on total scores, clustering and switching in Brazilian Portuguese-speaking subjects. Braz J Med Biol Res. 2004 Dec;37(12):1771–7.

17. Machado TH, Fichman HC, Santos EL, Carvalho VA, Fialho PP, Koenig AM, et al. Normative data for healthy elderly on the phonemic verbal fluency task - FAS. Dement Neuropsychol. 2009;3(1):55–60.

18. Bertoux M, Volle E, Funkiewiez A, de Souza LC, Leclercq D, Dubois B. Social Cognition and Emotional Assessment (SEA) is a marker of medial and orbital frontal functions: a voxel-based morphometry study in behavioral variant of frontotemporal degeneration. J Int Neuropsychol Soc. 2012 Nov;18(6):972–85.

19. Mariano LI, Caramelli P, Guimarães HC, Gambogi LB, Moura MVB, Yassuda MS, et al. Can Social Cognition Measurements Differentiate Behavioral Variant Frontotemporal Dementia from Alzheimer’s Disease Regardless of Apathy? J Alzheimers Dis. 2020;74(3):817–27.

20. Akoglu H. User’s guide to correlation coefficients. Turk J Emerg Med. 2018 Aug 7;18(3):91–3.

21. Mastrich Z, Hernandez I. Results everyone can understand: A review of common language effect size indicators to bridge the research-practice gap. Health Psychol. 2021 Oct;40(10):727–36.
